# High Mean Water Vapour Pressure Promotes the Transmission of Bacillary Dysentery

**DOI:** 10.1371/journal.pone.0124478

**Published:** 2015-05-06

**Authors:** Guo-Zheng Li, Feng-Feng Shao, Hao Zhang, Chun-Pu Zou, Hui-Hui Li, Jue Jin

**Affiliations:** 1 Data Center of Traditional Chinese Medicine, China Academy of Chinese Medical Sciences, Beijing, China; 2 Department of Control Science and Engineering, Tongji University, Shanghai, China; 3 School of Basic Medicine, Shanghai University of Traditional Chinese Medicine, Shanghai, China; The University of Tokyo, JAPAN

## Abstract

Bacillary dysentery is an infectious disease caused by *Shigella dysenteriae*, which has a seasonal distribution. External environmental factors, including climate, play a significant role in its transmission. This paper identifies climate-related risk factors and their role in bacillary dysentery transmission. Harbin, in northeast China, with a temperate climate, and Quzhou, in southern China, with a subtropical climate, are chosen as the study locations. The least absolute shrinkage and selectionator operator is applied to select relevant climate factors involved in the transmission of bacillary dysentery. Based on the selected relevant climate factors and incidence rates, an AutoRegressive Integrated Moving Average (ARIMA) model is established successfully as a time series prediction model. The numerical results demonstrate that the mean water vapour pressure over the previous month results in a high relative risk for bacillary dysentery transmission in both cities, and the ARIMA model can successfully perform such a prediction. These results provide better explanations for the relationship between climate factors and bacillary dysentery transmission than those put forth in other studies that use only correlation coefficients or fitting models. The findings in this paper demonstrate that the mean water vapour pressure over the previous month is an important predictor for the transmission of bacillary dysentery.

## Introduction

Bacillary dysentery (BD) is an infectious disease caused by Shigella dysenteriae that exhibits symptoms such as fever, abdominal pain, tenesmus and stool containing pus and blood [1]. The infection can be transmitted by the faecal-oral route via food, by person-to-person contact or through contaminated water [2]. BD outbreaks occur in areas with inadequate sanitation, and most cases occur during the summer and autumn. The incidence of BD has been elevated for many years. Shigella dysenteriae is listed as a Category B infectious agent in China and is also regarded as a global health problem by the World Health Organization (WHO).

The transmission of infectious diseases is determined by many factors, including social, economic and ecological conditions, access to health care, and intrinsic human immunity [3]. Meteorological factors, such as temperature, rainfall and relative humidity, may have an effect on the transmission of enteric infections, and as a type of enteric infection, BD is no exception. Many vectors, infectious agents and pathogen replication rates are particularly sensitive to climatic factors [4]. The replication rates of Shigella, the pathogenic bacteria that causes BD, have been demonstrated to be associated with meteorological factors.

In the past few years, many papers have investigated the relationship between climate factors and outbreaks of BD in Europe [5, 6], Australia [7], Peru [8], and China [9]. This relationship could provide new prevention methods and even result in the control of BD. However, the effects of climate factors on BD transmission that were found in previous studies are inconsistent. For example, a recent study found that the weekly number of infectious gastroenteritis cases increases by 8% for every 1°C increase in the average temperature [10].

In another recent study, Zhang et al. used regression analysis and found increases of 11%–16% in the number of cases of BD for each 1°C temperature increase [11]. The explanation for their findings was that the temperature had been elevated for a long time, which can not only reduce the body’s resistance, but also lead to air and water pollution, as well as to the replication of bacteria and viruses, as well as changes in the distribution of infectious diseases in crowds. High temperatures would lead to gastrointestinal function disorders, and under these conditions, dysentery bacilli may easily invade the intestines. One report shows that in a certain area, if relative humidity remains at a high level and the monthly mean minimum temperature remains at > 20°C, diarrhoeal intestinal infectious disease epidemic or outbreak is likely [12, 13].

In addition to temperature, climatic factors such as rainfall, relative humidity, and air pressure have been found to contribute to the changes in BD incidence [14]. Low levels of rainfall have been found to be associated with a high incidence of diarrhoea [15], while some studies have found that rainfall does not affect the transmission of specific BD strains [11]. The relationship between the incidence of BD and humidity cannot be ignored. Microbial metabolism and material exchange require certain amounts of water. The microorganism cannot live in an environment without proper humidity. Therefore, specific humidity conditions are necessary for the replication and spread of biological pathogens.

The varying results of previous studies may be due to the various local climate conditions, populations and ecological characteristics of the different regions. On the other hand, the validity of the methods used in these studies cannot be ignored. For example, some studies simply calculated the correlation coefficients between the BD incidence rate and climate factors and regarded those climate factors with high coefficients as being relevant for BD transmission. However, high coefficients do not suggest a strong relationship, and these methods also ignore the interaction effects between climate factors.

In this paper, the least absolute shrinkage and selectionator operator (LASSO), a feature selection method, is used to select relevant climate factors for BD transmission. Because LASSO can select relevant factors by building a linear regression model, the use of this operator explains the selected factors. Based on the selected factors and incidence rates of BD, an AutoRegressive Integrated Moving Average (ARIMA) model is built to predict the incidence rates of BD. The Mean Square Error (*MSE*) is applied as a valuation criterion. Through the feature selection stage, the mean water vapour pressure of the previous month is found to be strongly correlated with BD transmission, and ARIMA functions work well with this input.

## Materials and Methods

### Study Locations

In this study, we characterise the dynamic temporal trend of BD and identify climate-related risk factors and their roles in BD transmission. In the experiments, the cities of Harbin and Quzhou are chosen as the application sites of the study methods proposed in this paper.

**Fig 1:**
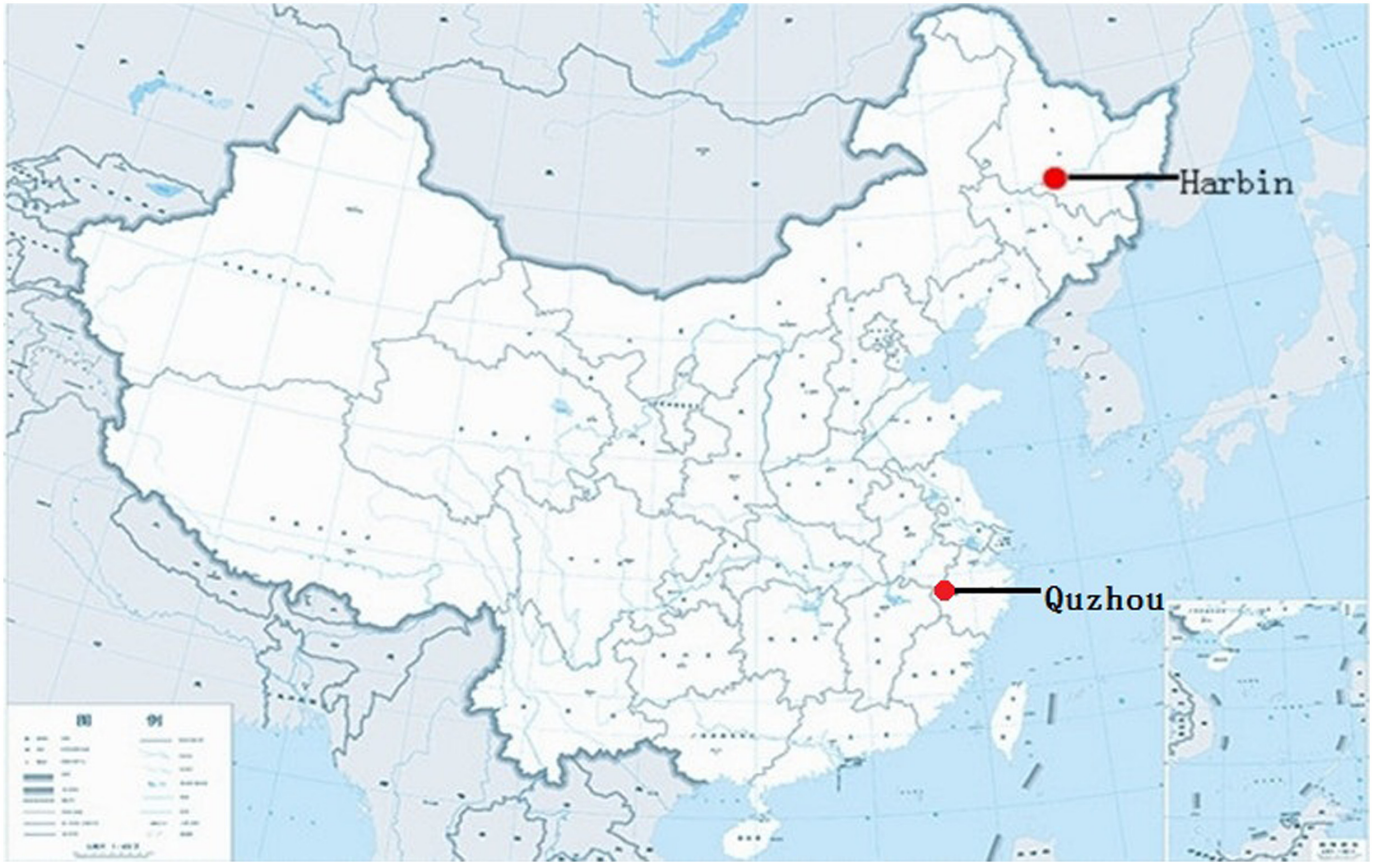
The red dots indicate the locations of Harbin and Quzhou in China.

Harbin is in northeast China, located between latitudes 44°04’ and 46°40’ north and longitudes 125°42’ and 130°10’ east, as shown in Fig 1. Harbin is a distinct city that has four seasons, with dry winters and wet, hot summers. The annual temperature ranges from -30 to +36°C and the annual mean temperature is approximately 3.5°C. The annual rainfall typically measures between 400 and 600 mm.

Quzhou is in southern China, located between latitudes 28°14’ and 29°30’ north and longitudes 118°01’ and 119°20’ east, as shown in Fig 1. Quzhou has a low level of rainfall in the winter and a hot summer with high temperatures and rainfall. The mean annual temperature ranges from 16.3 to 17.3°C, and the annual rainfall measures approximately 1843 mm.

### Data Collection

In this study, records on BD cases in Harbin from 2002 to 2008 and Quzhou from 1999 to 2005 are obtained from the National Notifiable Disease Surveillance System. The disease surveillance data used in this study were previously identified. All BD cases are first diagnosed symptomatically.

Monthly climate data of both cities are collected from the China Meteorological Data Sharing Service System (http://cdc.cma.gov.cn/home.do). The data set is compiled from national surface information-based monthly reports submitted from each province in China, and these data sets follow the rules of statistical methods for national surface climate data and surface weather observing regulations. The details of the climate factors are as follows.

- monthly mean temperature (MT)
- monthly mean maximum temperature (MaxT)
- monthly mean minimum temperature (MinT)
- monthly extreme maximum temperature (EMaxT)
- monthly extreme minimum temperature (EMinT)
- monthly mean air pressure (MAP)
- monthly extreme maximum air pressure (EMaxAP)
- monthly extreme minimum air pressure (EMinAP)
- monthly mean relative humidity (RH)
- monthly mean minimum relative humidity (MinRH)
- monthly accumulative precipitation (AP)
- daily maximum precipitation (DMaxP)
- monthly mean wind velocity (MWV)
- monthly maximum wind velocity (MaxWV)
- monthly minimum wind velocity (MinWV)
- monthly sunshine duration (SD)
- monthly mean water vapour pressure (MWVP)

### Statistical Analyses

The collected data in this study contain monthly climate factors and BD incidence rates. Because the causes of BD transmission are unknown, predicting the incidence rate for one month will require the use of more than the current month’s climate factors. In this study, climate factors over a period of three months are applied to predict the incidence rate for a single month. The related climate factors are selected based on their performance in the analysis. The selected factors are then applied to the prediction model.

#### Feature Selection

The main purpose of this study is to determine the climate factors that are related to the BD epidemic and to build a prediction model. One popular algorithm, LASSO, is applied to select the related climate factors [16]. LASSO, which was originally proposed for linear regression models, has become a popular method for model selection and shrinkage estimation. The main idea of LASSO is as follows.

Suppose we have data (*x*_*i*_, *y*_*i*_), i = 1, 2…N, where *y*_*i*_ are the responses and *x*_*i*_ = (*x*_*i*1_, *x*_*i*2_, … *x*_*ip*_)^*T*^ are the predictor variables. A hypothesis is that both predictor variables and responses are independent, and *x*_*ij*_ are standardized, then $\sum_{i}x_{ij}/N=0,\sum_{i}x_{ij}^{2}/N=1$.

$$\begin{array}{c} L=\sum_{i}{\left(y_{i}-\sum_{p}\beta_{p}x_{ip}\right)}^{2}+\lambda \sum_{p}{∥\beta_{p}∥}_{1}, \end{array}$$

where *β*_*p*_ denotes the regression coefficient of the *pth* feature. The norm-1 regularised *λ*∑_*p*_‖*β*_*p*_‖_1_ in LASSO typically leads to a sparse solution in the feature space, which means that the regression coefficients for the most irrelevant or redundant features are shrunk down to zero. In *λ*∑_*p*_‖*β*_*p*_‖_1_, *λ* is a nonnegative regularization parameter that is related to the regression coefficients. The LASSO continuously shrinks the regression coefficients towards 0 as *λ* increases. If *λ* is sufficiently large, some regression coefficients are shrunk down to exactly 0. Moreover, the prediction accuracy can be improved by continuous shrinkage due to the bias-variance trade-off.

#### Evaluation Criterion

In this study, the Mean Square Error (*MSE*) is used as an evaluation criterion for feature selection. *MSE* is a popular criterion used to evaluate the performance of an estimator or a predictor [17]. Suppose $\hat{Y}$ is a vector containing n predictions and *Y* is a vector containing n true values. Then, the *MSE* of the predictor is described as follows:

$$\begin{array}{c} MSE=\frac{1}{n}\sum_{i=1}^{n}{\left(\overset{\wedge }{Y_{i}}-Y_{i}\right)}^{2}. \end{array}$$

The smaller the *MSE*, the better the predictor is.

#### Details of Feature Selection Stage

In our experiment, the ability of LASSO is assessed by a 10-fold cross validation. To identify climate factors related to the BD epidemic, we set two threshold values; *θ*_1_ and *θ*_2_. In every fold cross validation, the optimal regression coefficients are selected according to the validation set. If the regression coefficient of a feature is less than *θ*_1_, the coefficient would be set to zero; otherwise, the coefficient would be set to one. After the 10-fold cross validation, every feature has ten coefficients containing zeros and ones. If the number of zeros in a feature’s coefficients is larger than *θ*_2_, that feature would be removed from the feature set. The remaining features would be processed as described above, and this process would be repeated *N* times. When the iteration is completed, the remaining feature set contains the optimal climate factors for the regression model.

### Regression Analysis

The AutoRegressive Integrated Moving Average (ARIMA) model is famous for describing and forecasting epidemic prevalence with superior accuracy and practicability [18]. An ARIMA model is defined as an ARIMA(*p*, *d*, *q*)(*P*, *D*, *Q*) in which the first parenthesis holds the non-seasonal autoregressive (*p*), differencing (*d*), and the moving average (*q*) orders [19], and the second parenthesis holds their seasonal counterparts. These terms are determined by an autocorrelation function (ACF) and a partial autocorrelation function (PACF) [20].

## Results

### The temporal distribution of BD in Harbin and Quzhou

**Fig 2:**
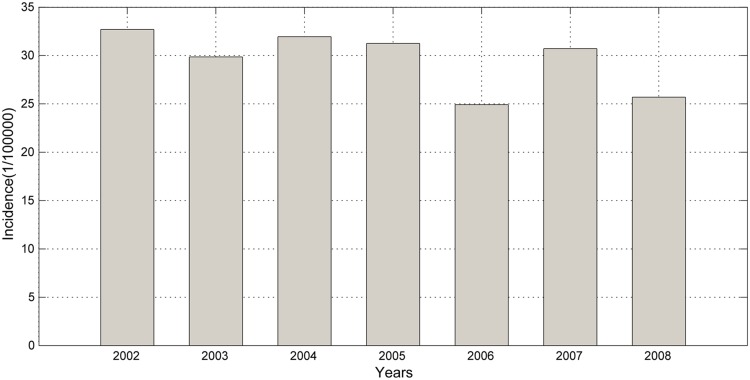
Annual incidence of BD from 2002 to 2008 in Harbin.

**Fig 3:**
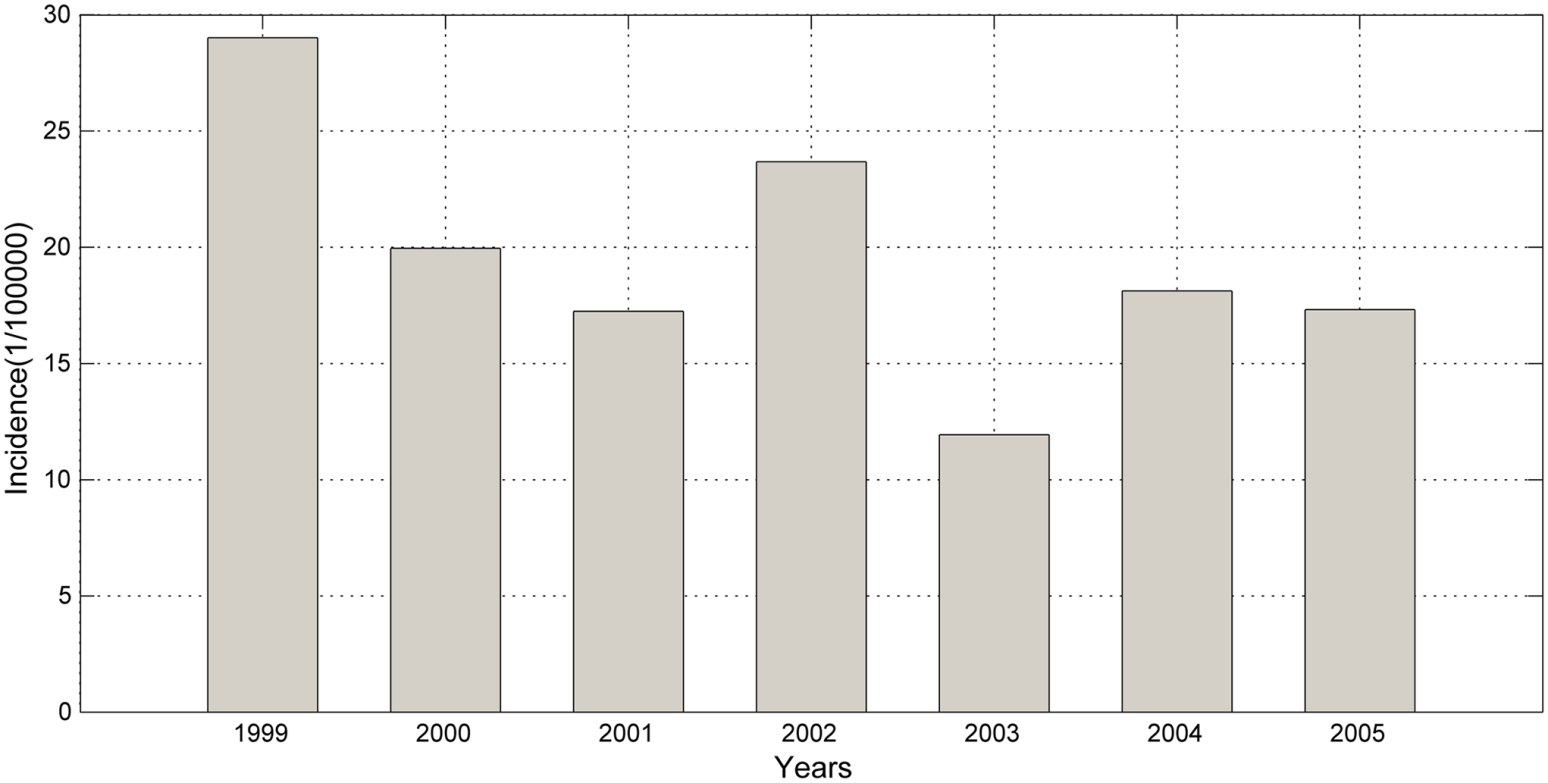
Annual incidence of BD from 1999 to 2005 in Quzhou.

A total of 20,256 cases of BD were reported in Harbin from 2002 to 2008. As shown in Fig 2, the annual BD incidence varied during this period. The highest incidence occurred in 2002 with 32.69 cases per 100,000 residents, and the lowest occurred in 2006 with 24.91 cases per 100,000 residents. In Quzhou, there were 3,370 cases reported from 1999 to 2005; the highest incidence occurred in 1999 (29 cases) and the lowest occurred in 2003 (11.93 cases), as shown in Fig 3.

**Fig 4:**
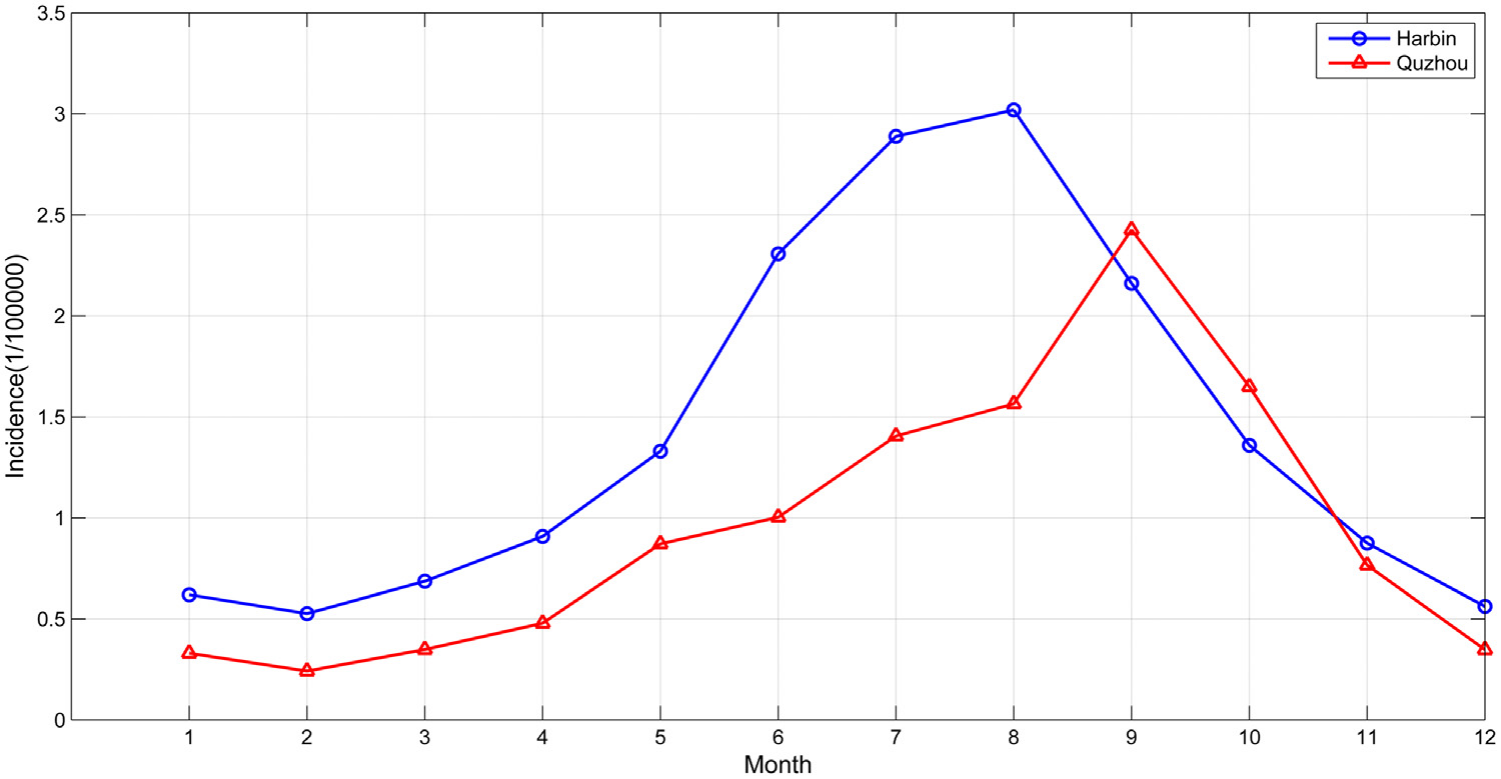
Monthly distribution of BD in Harbin and Quzhou.

An obvious periodicity by season is observed (Fig 4). The epidemic incidence rates peaked every summer and autumn (June to October) of the years studied.

### Analysing the relationship between BD incidence and climate factors

To identify the climate factors causing the BD epidemic, one-month, two-month, three-month, four-month and five-month climate data are used. The method is described in the Methods Section. In this paper, “one-month” refers to the specific month for which incidence is predicted using the same month’s climate data; “two-month” refers to the use of climate data from the current and the past one month to predict the incidence; “three-month” refers to the use of the current and the past two months’ climate data to predict incidence; and the trend is similar for “four-month” and “five-month”.

**Fig 5:**
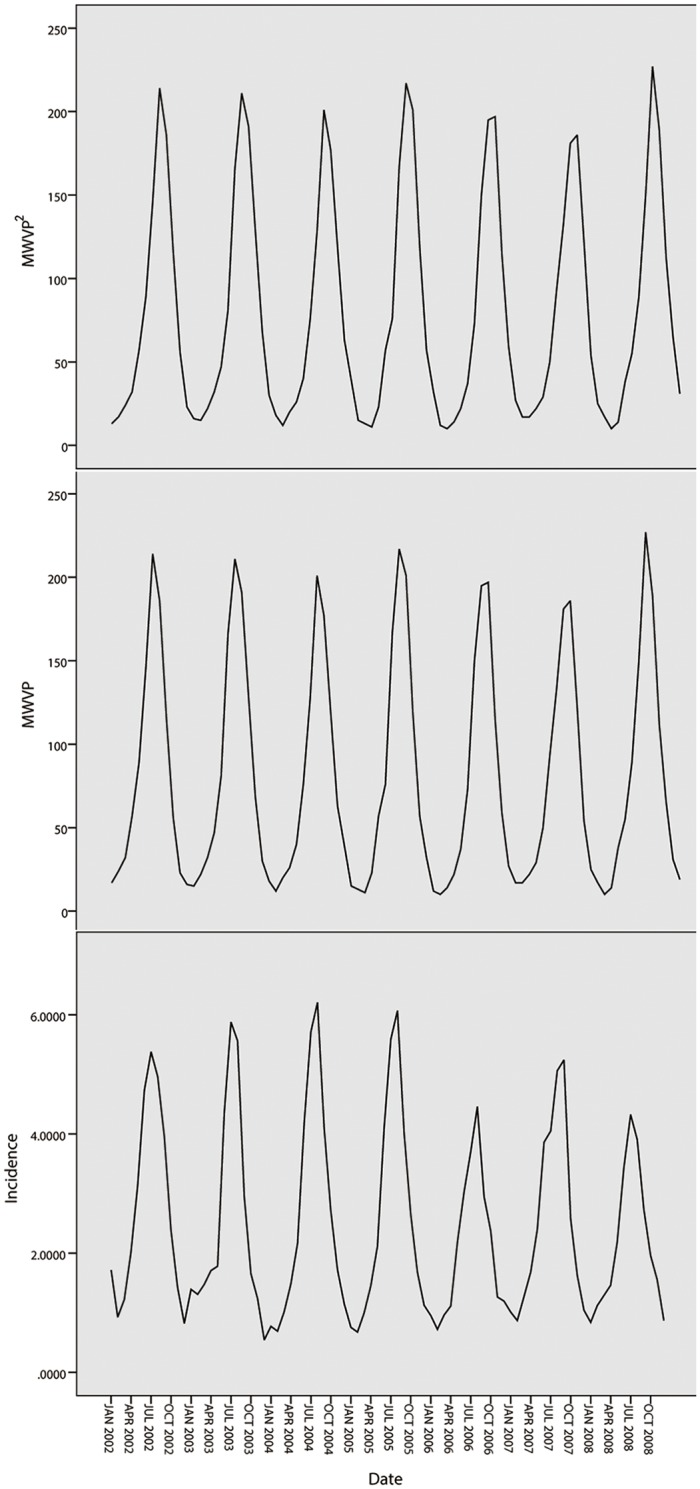
Tendencies of BD incidence, MWVP and MWVP^2^ in Harbin.

**Fig 6:**
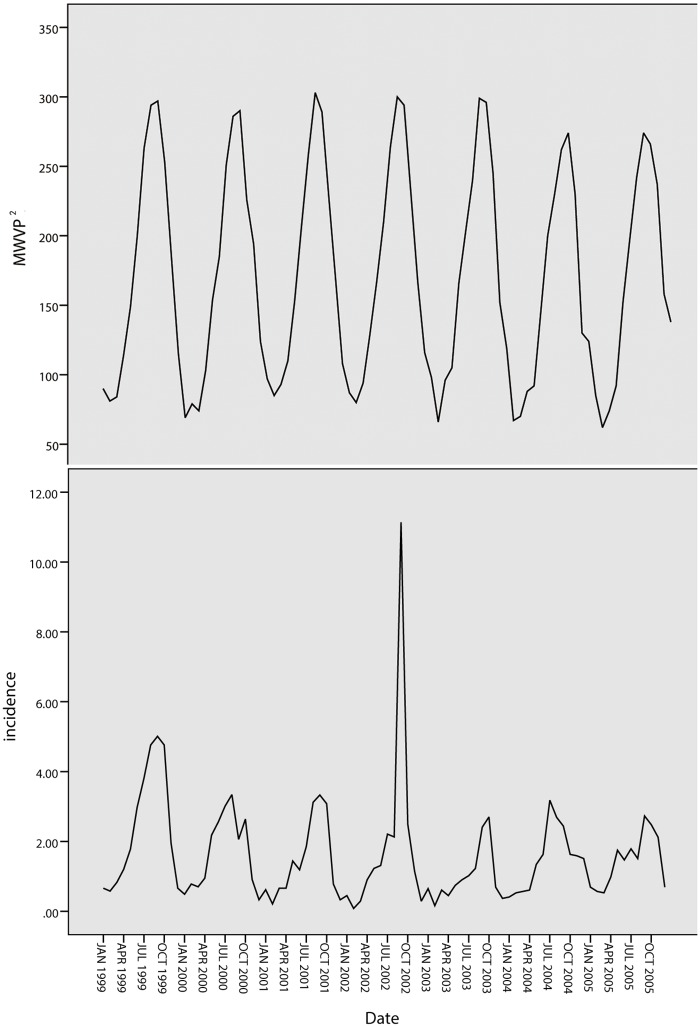
Tendencies of BD incidence and MWVP^2^ in Quzhou.

The ultimate *MSE* in Harbin and Quzhou and the selected climate factors of feature selection using five different data sets is described in Table 1. As shown in this table, the ultimate *MSE* is lowest when three-month climate data are used for Harbin and two-month climate data are used for Quzhou. This finding indicates that BD transmission is not only related to the current month’s climate factors but also to the past months’ factors. As shown in Table 1, the errors of the prediction models are minimal when MWVP and MWVP^2^ are used for Harbin and MWVP^2^ is used for Quzhou. This finding indicates that MWVP^2^ has been selected as the most important factor for both cities. Table 1 also shows that the same results are seen when the three-month, four-month and five-month climate data are used. Therefore, there is no need to use six-month data or more to study the relationship between BD and climate factors. In Fig 5, the tendencies of BD incidence in Harbin, the MWVP and the MWVP^2^ are described. Fig 6 describes the tendencies of BD incidence and the MWVP^2^. In Fig 5, the BD incidence, MWVP and MWVP^2^ show consistently changing trends, and the BD incidence and MWVP^2^ show the same in Fig 6.

**Table 1:** *MSE* and selected climate factors of feature selection using five different data sets.

	City	One-month	Two-month	Three-month	Four-month	Five-month
*MSE*	Harbin	0.282	0.253	0.251	0.257	0.254
	Quzhou	0.563	0.529	0.531	0.530	0.534
Selected factors	Harbin	MWVP	MWVP, MWVP^2^	MWVP, MWVP^2^	MWVP, MWVP^2^	MWVP, MWVP^2^
	Quzhou	MAP, MinT	MWVP^2^	MWVP^2^	MWVP^2^	MWVP^2^

MWVP^2^ means the MWVP over the previous month.

### Building the prediction model

Based on the selected climate factors and BD incidence, ARIMA is applied to build the prediction model. The analysis is performed using the SPSS software with a significance level of 0.05.

#### Prediction model for Harbin

In this section, the data from Harbin are used by ARIMA to build the prediction model. Data from January 2002 to June 2008 are used in the experiments as observations to build the estimation model.

**Fig 7:**
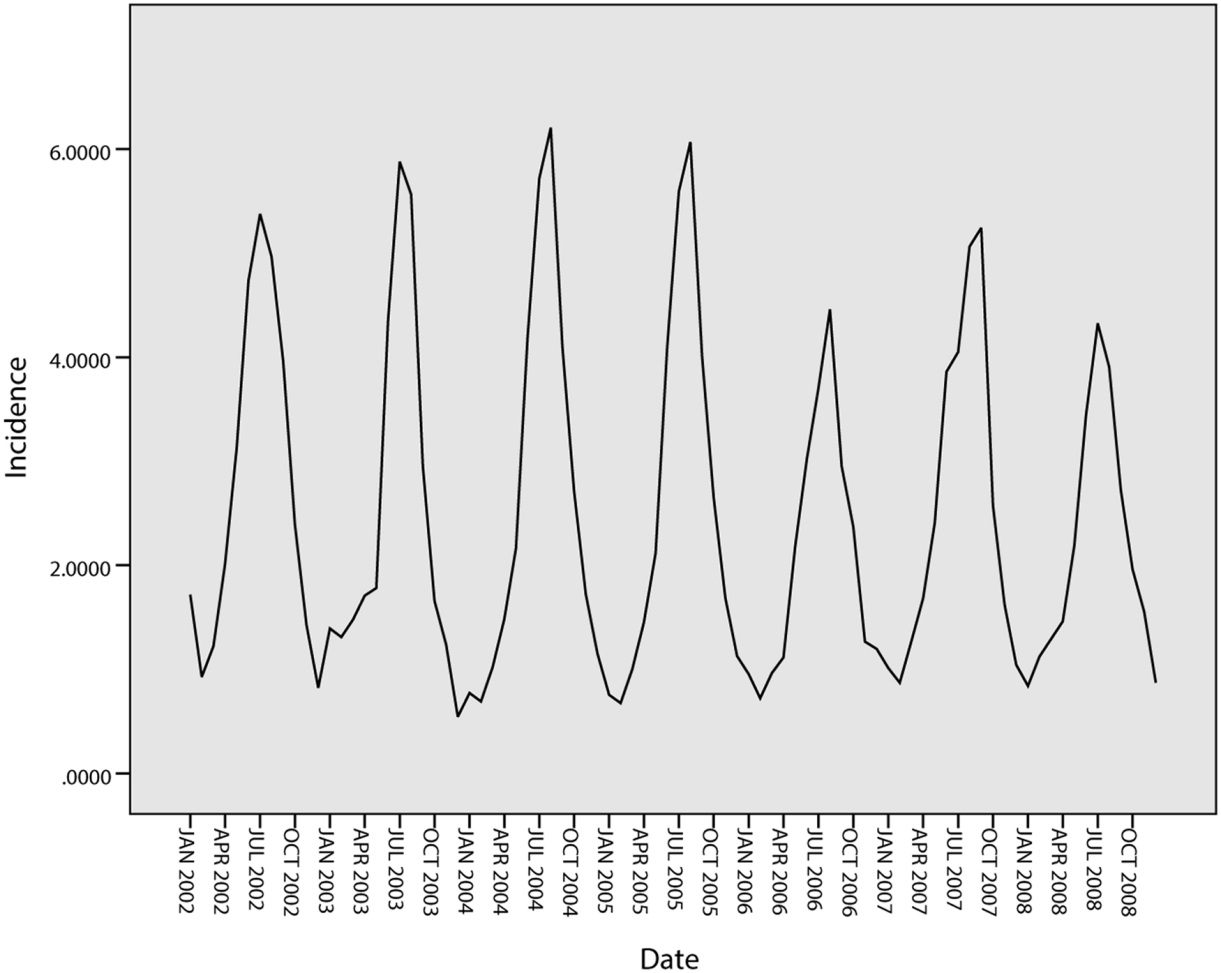
Tendencies of BD incidence in Harbin.

**Fig 8:**
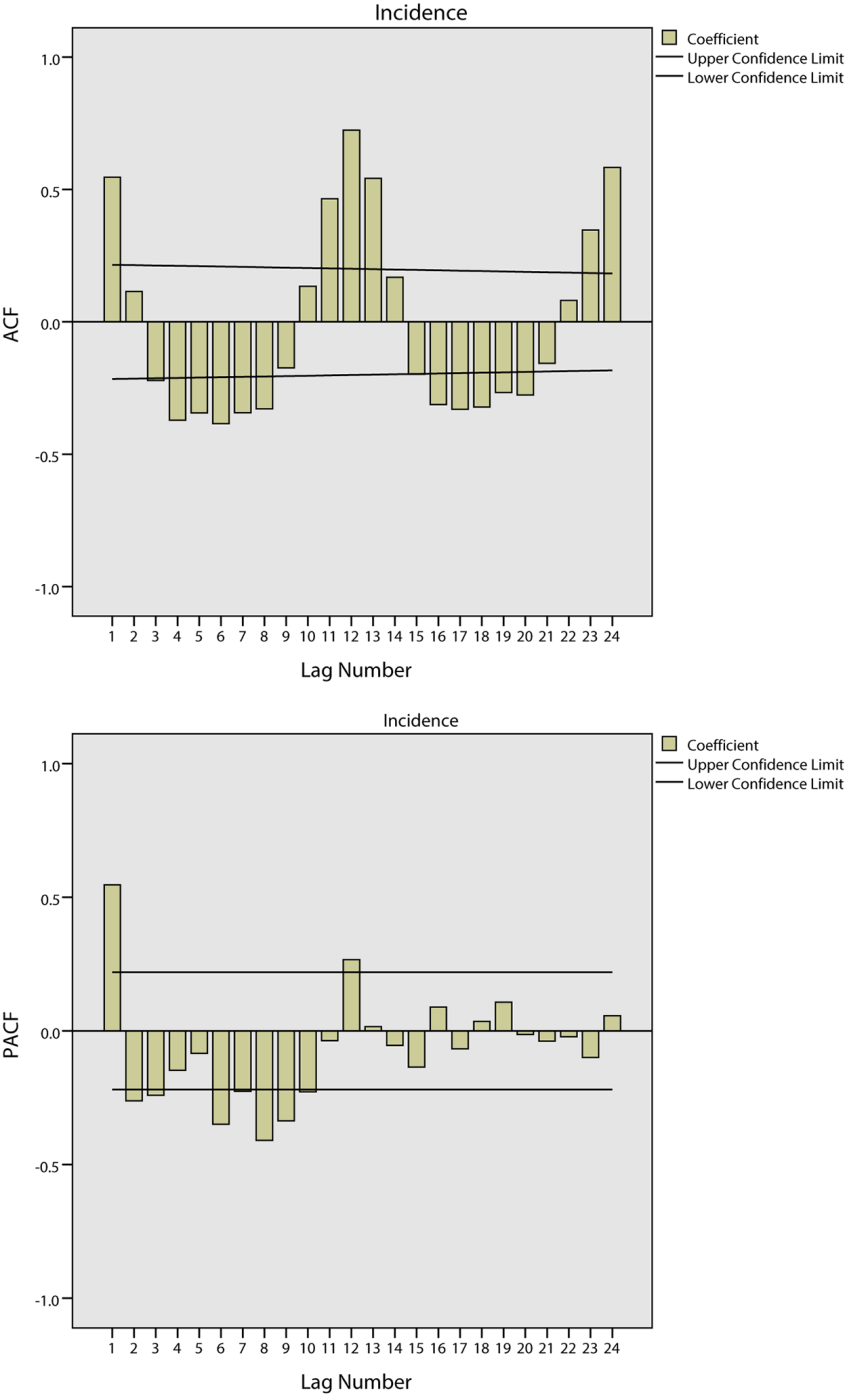
Results of ACF and PACF for time series analysis using 1-step non-seasonal difference in Harbin.

**Fig 9:**
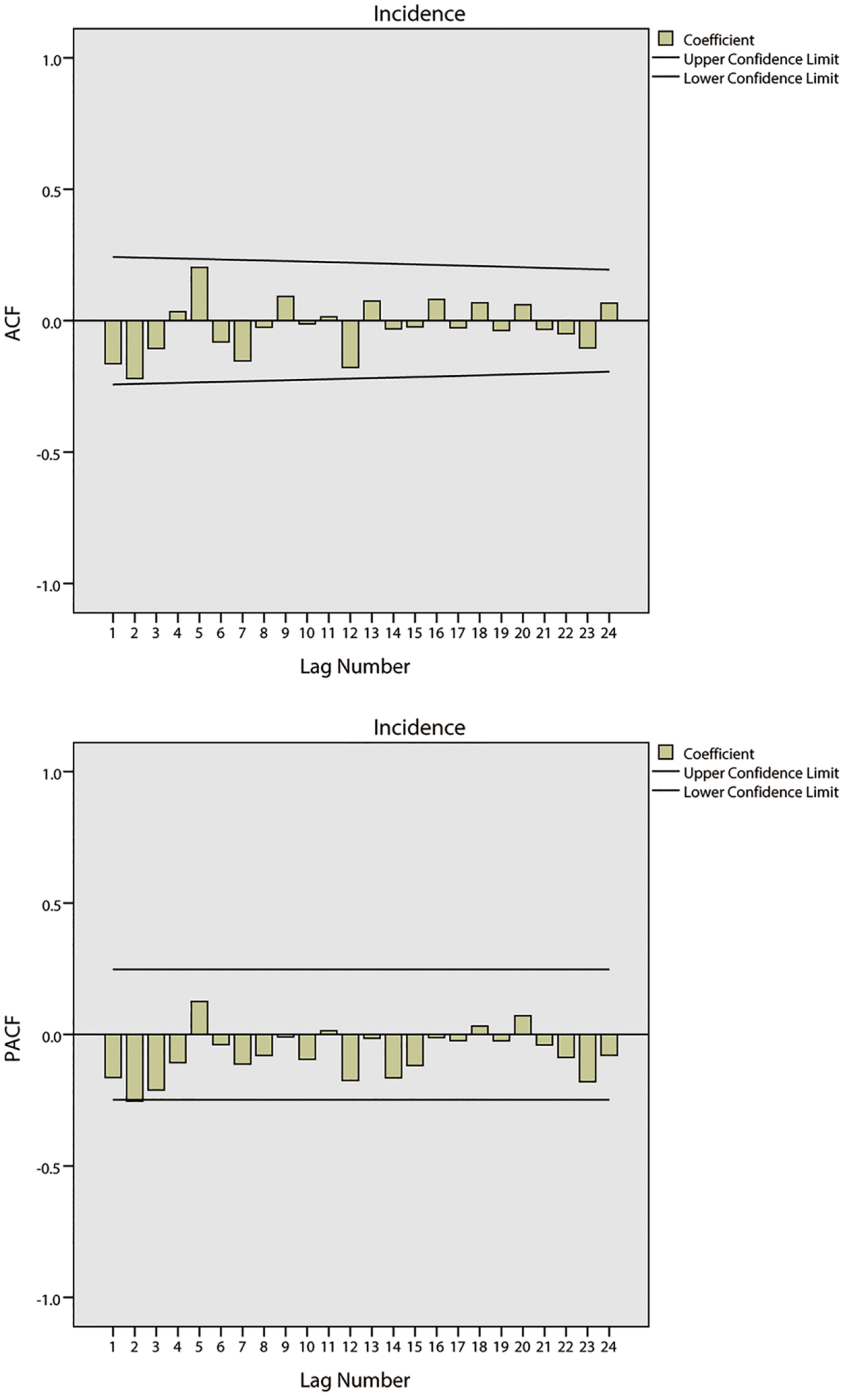
Results of ACF and PACF for time series analysis using 1-step non-seasonal and 1-step seasonal difference in Harbin.

Fig 7 shows the tendencies of the BD incidence in Harbin from 2002 to 2008. The series shows a non-stationary mean, and the mean of the BD incidence is therefore stabilized by differences. Firstly, a 1-step non-seasonal difference is applied in the ARIMA model. The ACF and PACF of the BD incidence are shown in Fig 8. As this figure demonstrates, the BD incidence shows a strong seasonal association. Secondly, a 1-step seasonal difference is used; the ACF and PACF of the BD incidence are shown in Fig 9, where the ACF shows a quick decay that cuts off at lag 2, suggesting MA(*q* = 2), while the PACF cuts off at lag 3, which suggests AR(*p* = 3). Through the analysis of the fitted parameters of ARIMA(3,1,2)(0,1,0), the significance levels of AR(*p* = 1), AR(*p* = 2) and AR(*p* = 3) are 0.000, 0.000 and 0.968, respectively. Because the significance of AR(*p* = 3) is greater than 0.05, *p* = 2 is therefore chosen as the final parameter.

**Fig 10:**
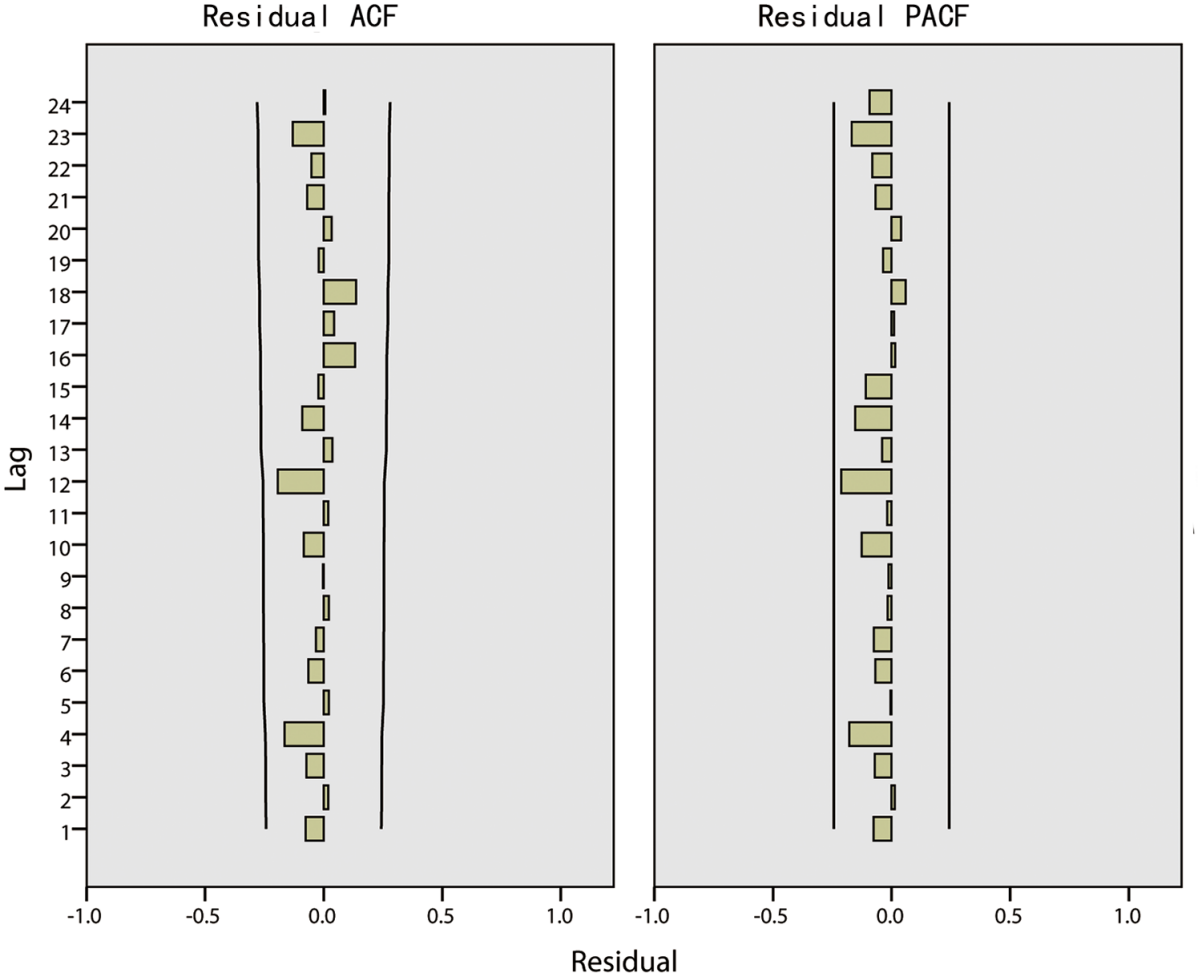
The ACF and PACF of the residual in Harbin.

Through the above analysis, the ARIMA model can be defined as ARIMA(2,1,2)(0,1,0). The fitted parameters of the ARIMA model are shown in Table 2 and Table 3. Table 2 describes the statistics in which the stationary R-squared is 0.175 and the significance of Ljung-Box is 0.686, which is greater than 0.05. As shown in Table 3, the significance levels of AR(*p* = 1), AR(*p* = 2), MA(*q* = 1) and MA(*q* = 2) are all less than 0.05, which means that *p* = 2 and *q* = 2 are suitable. The ACF and PACF of the residual are shown in Fig 10 where all of the coefficients are between -0.02 and 0.02. The statistics of the ACF and PACF of the residual are shown in Table 4 where all of the significance levels are greater than 0.05. According to the fitted statistics and parameters, it appears that the ARIMA(2,1,2)(0,1,0) model is reasonable.

**Table 2:** Statistics of ARIMA model in Harbin.

Model Fit statistics		Ljung-Box		
Stationary R-squared	Normalized BIC	Statistics	DF	Sig.
0.175	-0.416	11.004	14	0.686

**Table 3:** Parameters of ARIMA model in Harbin.

		Estimate	SE	t	Sig.
AR	Lag 1	0.533	0.239	2.232	0.030
	Lag 2	-0.758	0.183	-4.151	0.000
Difference		1			
MA	Lag 1	0.711	0.287	2.482	0.016
	Lag 2	-0.608	0.257	-2.368	0.021
Seasonal Difference		1			

**Table 4:** The ACF statistics of residual of ARIMA model in Harbin.

Lag	Autocorrelation	Std. Error	Box-Ljung Statistic		
			Value	df	Sig.
1	-0.061	0.121	0.255	1	0.614
2	0.009	0.120	0.261	2	0.878
3	-0.051	0.119	0.442	3	0.931
4	-0.187	0.118	2.935	4	0.569
5	0.063	0.117	3.220	5	0.666
6	-0.116	0.116	4.209	6	0.648
7	-0.020	0.115	4.239	7	0.752
8	0.023	0.114	4.279	8	0.831
9	-0.018	0.113	4.305	9	0.890
10	-0.069	0.112	4.681	10	0.911
11	0.013	0.111	4.694	11	0.945
12	-0.176	0.110	7.230	12	0.842
13	0.056	0.109	7.490	13	0.875
14	-0.096	0.108	8.272	14	0.875
15	-0.036	0.107	8.384	15	0.907
16	0.121	0.106	9.693	16	0.882
17	0.060	0.105	10.025	17	0.903
18	0.103	0.104	11.004	18	0.894
19	0.008	0.103	11.009	19	0.924
20	0.004	0.102	11.011	20	0.946
21	-0.047	0.101	11.225	21	0.958
22	-0.091	0.099	12.066	22	0.956
23	-0.143	0.098	14.180	23	0.922
24	0.027	0.097	14.257	24	0.941

**Fig 11:**
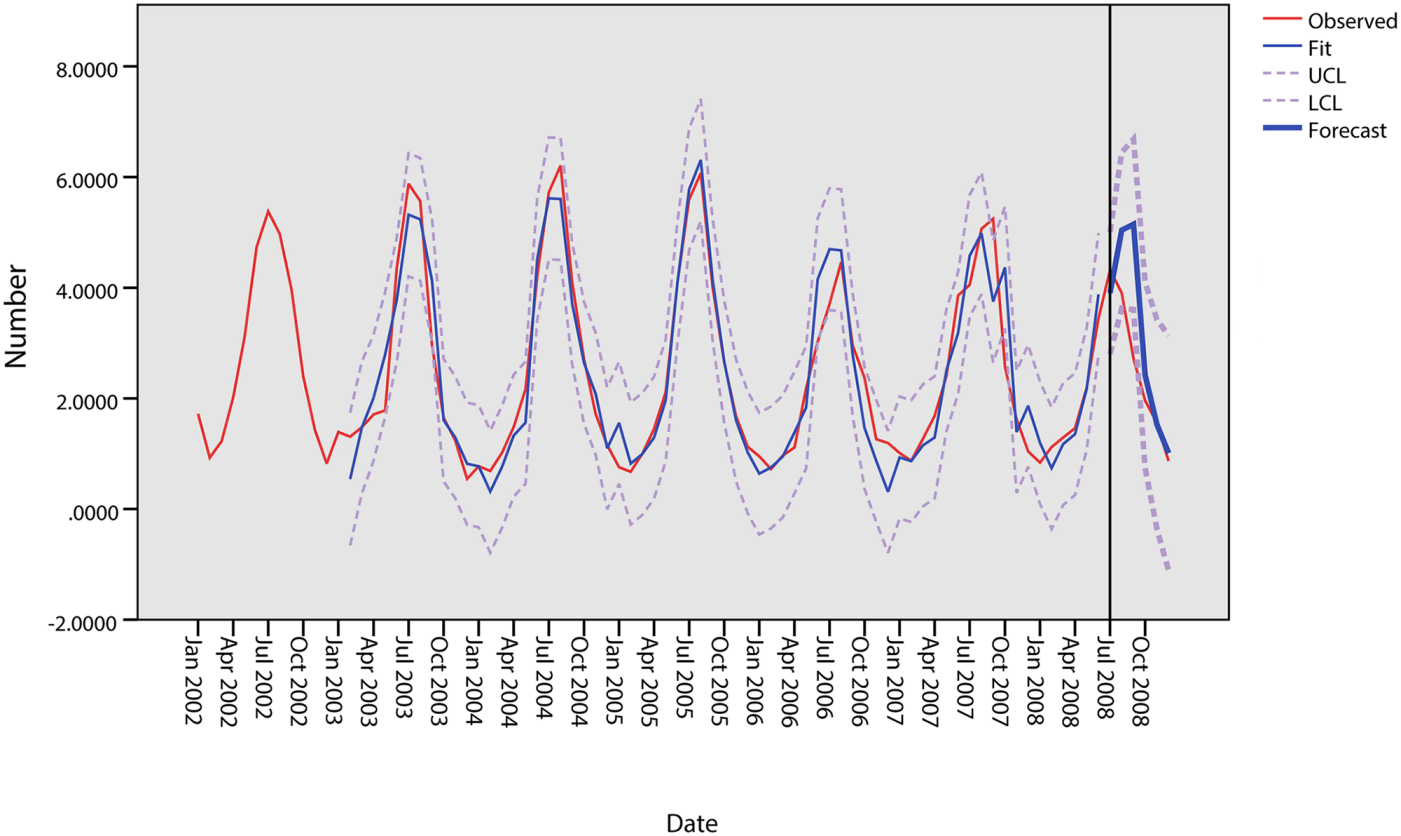
Results of the prediction model in Harbin.

Based on the ARIMA model, this study attempts to predict BD cases from July to December, 2008, and the result is shown in Table 5. The estimated and predicted results in Fig 11 show that the predicted data are similar to the observed data. The predicted monthly numbers of cases from July to December 2008 are 3.9869, 4.7722, 4.9359, 2.0726, 1.4967 and 0.7206, all of which fall within the confidence interval limits.

**Table 5:** Results of ARIMA forecasting BD incidence from July to December, 2008 in Harbin.

Observations	Observed	Forecast	UCL	LCL
July	4.33	3.99	5.13	2.85
August	3.90	4.77	6.25	3.30
September	2.72	4.94	6.55	3.32
October	1.96	2.07	3.82	0.33
November	1.55	1.50	3.45	-0.46
December	0.87	0.72	2.91	-1.47

Confidence Limit is 95%.

UCL: Upper Confidence Limits, LCL: Lower Confidence Limits.

##### Prediction model for Quzhou

In this section, the data from Quzhou are used by ARIMA to build the prediction model. Data from January 1999 to June 2005 are used in the experiments as observations to build the estimation model.

**Fig 12:**
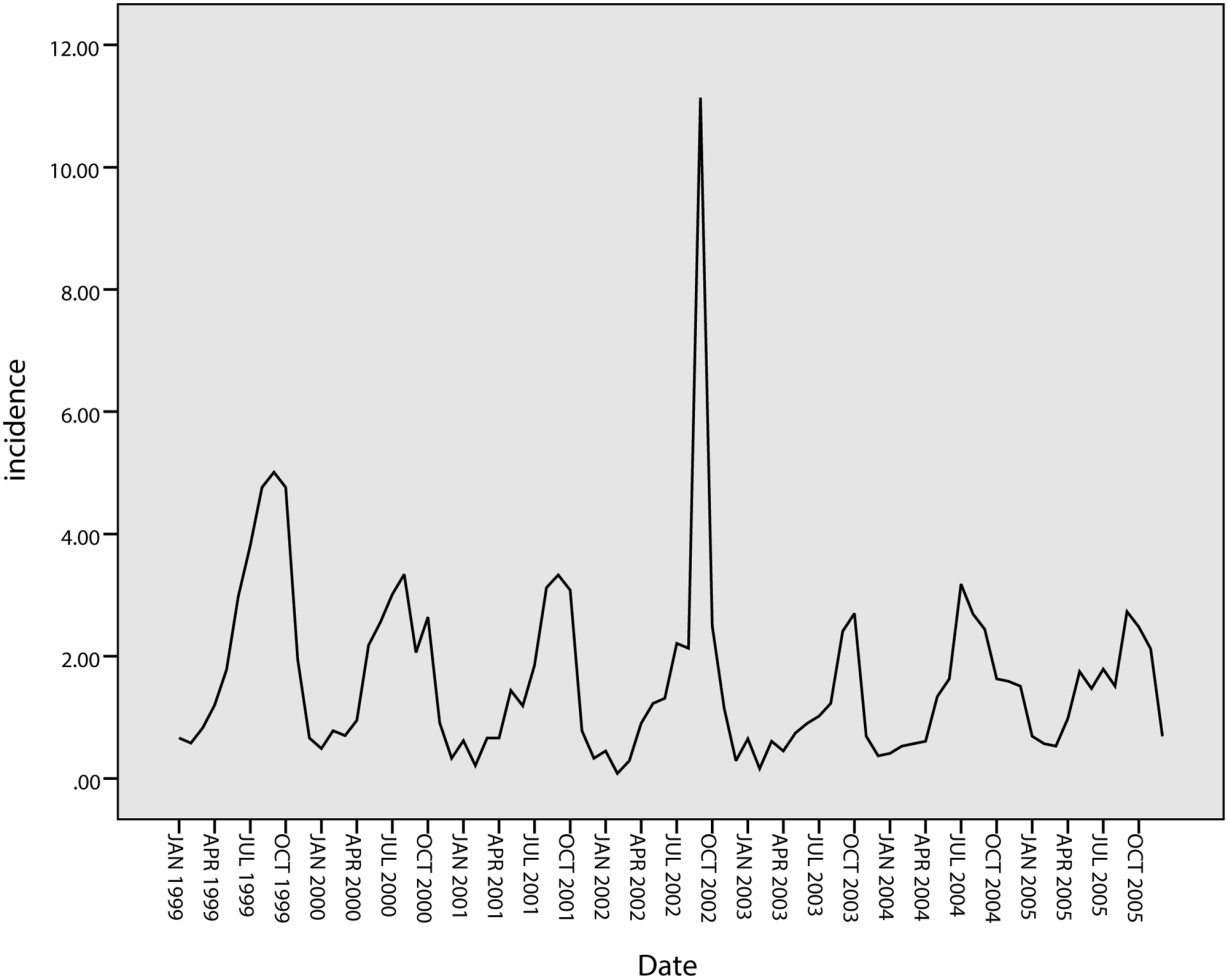
Tendencies of BD incidence in Quzhou.

**Fig 13:**
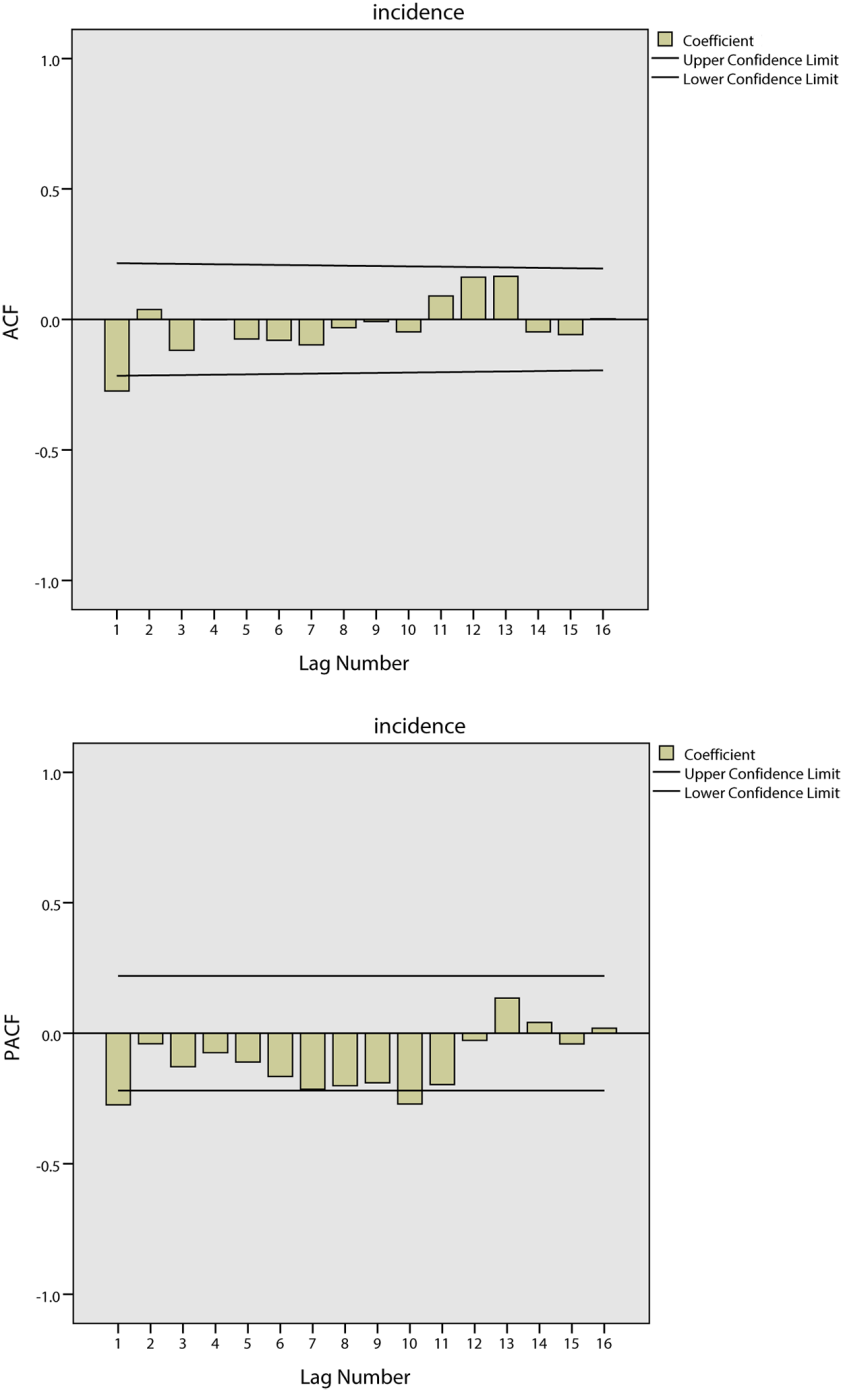
Results of ACF and PACF for time series analysis using 1-step non-seasonal difference in Quzhou.

**Fig 14:**
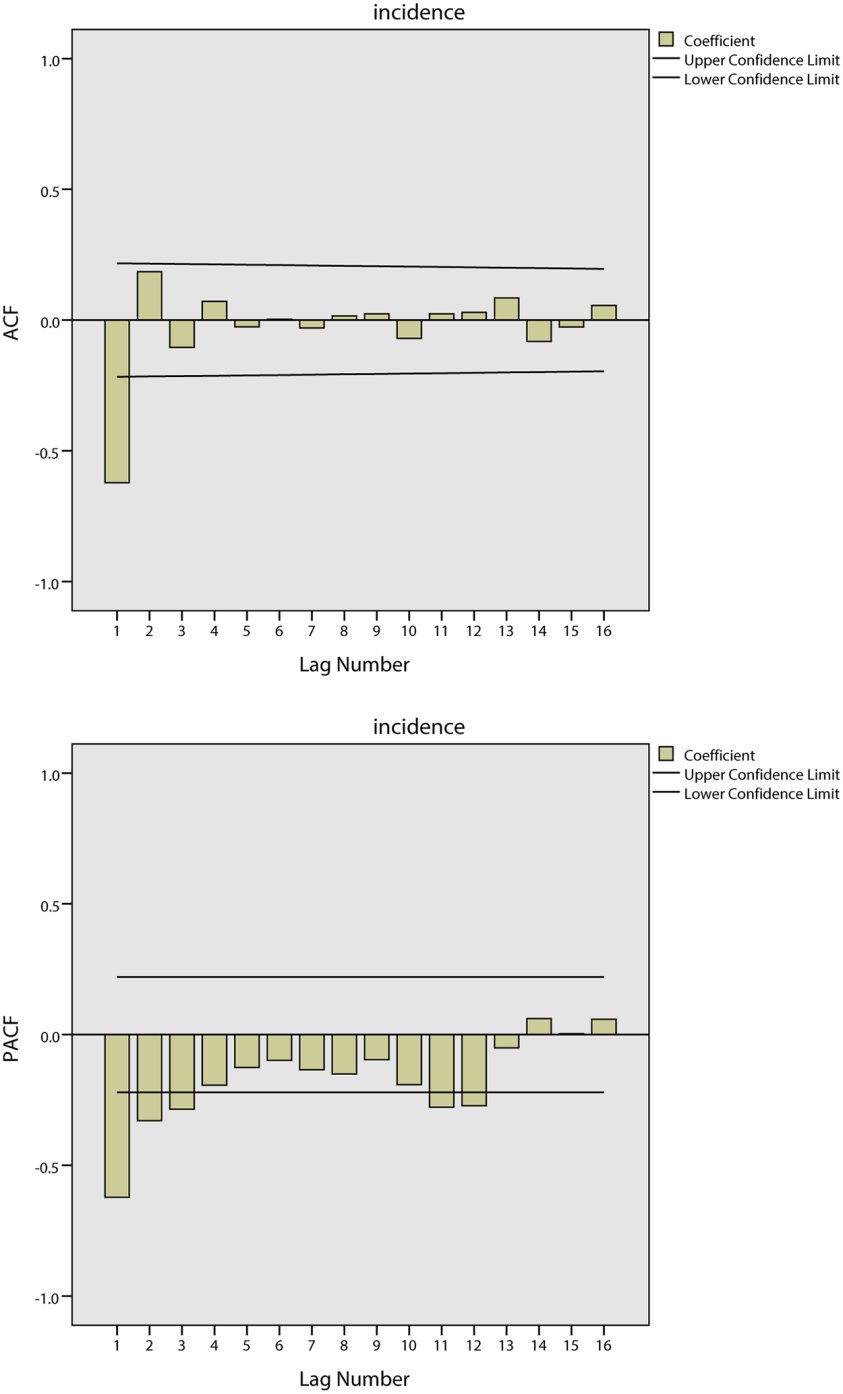
Results of ACF and PACF for time series analysis using 2-step non-seasonal difference in Quzhou.

**Fig 15:**
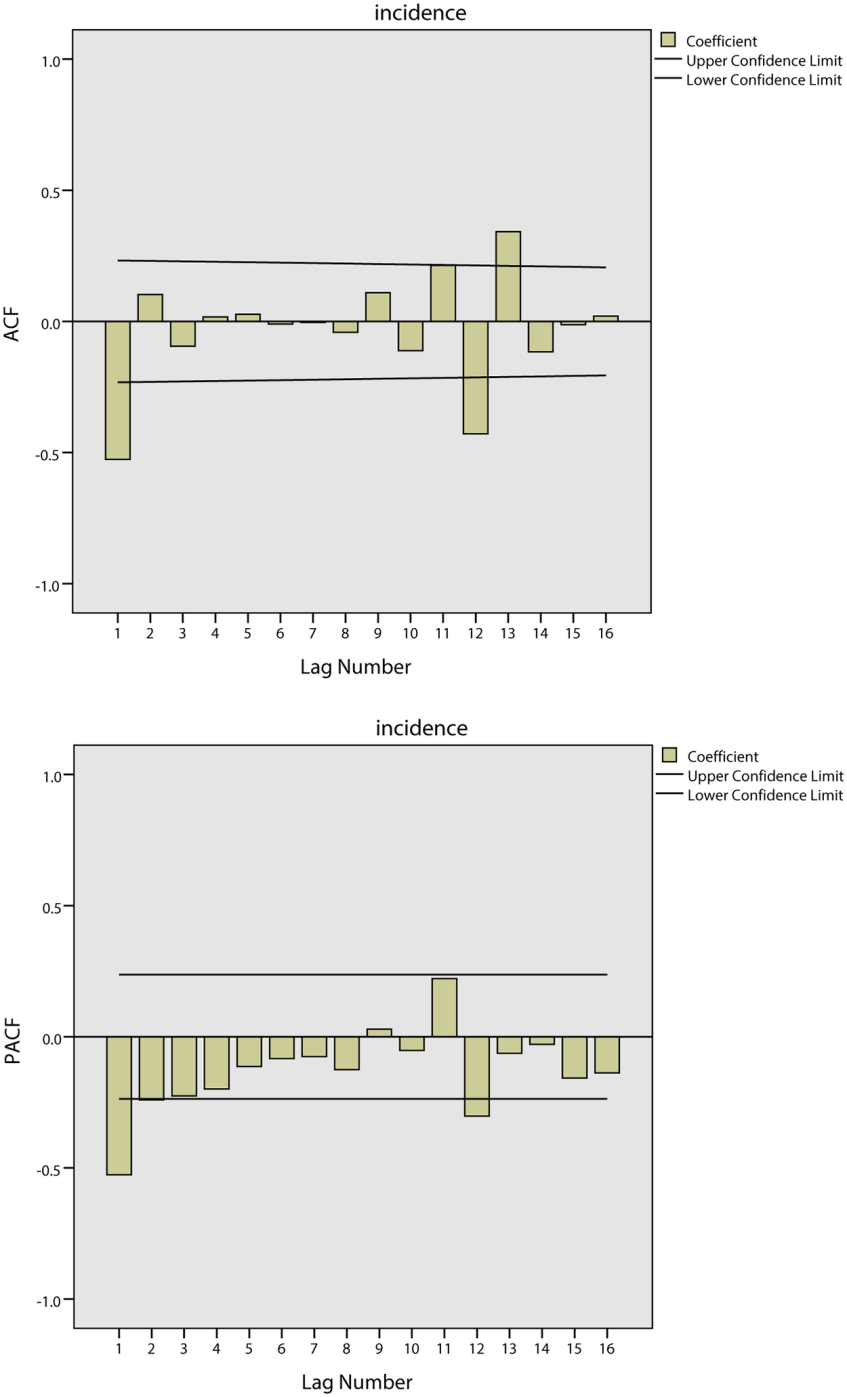
Results of ACF and PACF for time series analysis considering seasonal difference in Quzhou.

Fig 12 shows the tendencies of the BD incidence in Quzhou from 1999 to 2005. The series shows a non-stationary mean, and the mean of the BD incidence is stabilized by differences. Initially, both 1-step and 2-step non-seasonal differences are separately applied. The ACF and PACF of the BD incidence are shown in Fig 13 and Fig 14. Fig 13 demonstrates that neither the ACF nor the PACF decays quickly when using a 1-step non-seasonal difference. Judging by the results shown in Fig 14, in which a 2-step non-seasonal difference is applied, *p* = 3, *d* = 2 and *q* = 1 would be suitable. Considering the seasonal nature of the data, a 1-step seasonal difference is used, and Fig 15 describes the ACF and PACF of the BD incidence. Neither the ACF nor PACF demonstrates fast decay (Fig 15).

**Fig 16:**
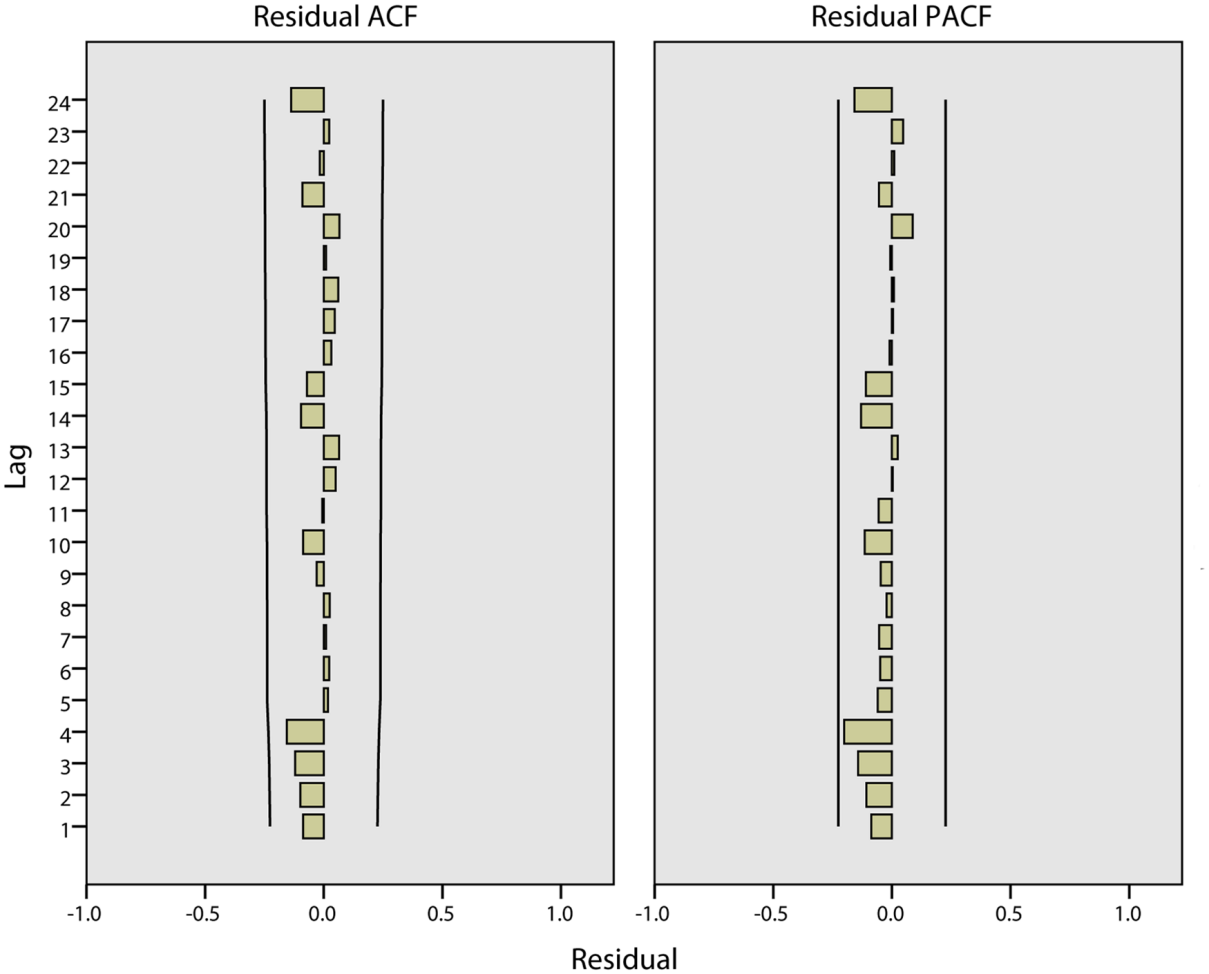
The ACF and PACF of the residual in Quzhou.

Through the above analysis, the prediction model can be defined as ARIMA(3,2,1)(0,0,0). The fitted parameters of the ARIMA model are shown in Table 6. The ACF and PACF of the residual are shown in Fig 16 where all of the coefficients are between -0.02 and 0.02. The statistics of the ACF and PACF of the residual are shown in Table 7, in which all of the significance levels are greater than 0.05. According to the fitted statistics and parameters, the ARIMA(3,2,1)(0,0,0) model is reasonable.

**Table 6:** Statistics of ARIMA model in Quzhou.

Model Fit statistics		Ljung-Box		
Stationary R-squared	Normalized BIC	Statistics	DF	Sig.
0.772	1.019	8.062	14	0.886

**Table 7:** The ACF statistics of residual of ARIMA model in Quzhou.

Lag	Autocorrelation	Std. Error	Box-Ljung Statistic		
			Value	df	Sig.
1	-0.087	0.113	0.591	1	0.442
2	-0.099	0.112	1.363	2	0.506
3	-0.121	0.112	2.534	3	0.469
4	-0.156	0.111	4.508	4	0.342
5	0.018	0.110	4.535	5	0.475
6	0.023	0.109	4.580	6	0.599
7	0.010	0.109	4.588	7	0.710
8	0.025	0.108	4.642	8	0.795
9	-0.030	0.107	4.723	9	0.858
10	-0.087	0.106	5.398	10	0.863
11	-0.006	0.105	5.401	11	0.910
12	0.050	0.104	5.633	12	0.933
13	0.065	0.104	6.022	13	0.945
14	-0.095	0.103	6.881	14	0.939
15	-0.071	0.102	7.364	15	0.947
16	0.032	0.101	7.464	16	0.963
17	0.047	0.100	7.680	17	0.973
18	0.061	0.099	8.062	18	0.978
19	0.009	0.098	8.071	19	0.986
20	0.066	0.098	8.531	20	0.988
21	-0.089	0.097	9.385	21	0.986
22	-0.017	0.096	9.414	22	0.991
23	0.023	0.095	9.473	23	0.994
24	-0.138	0.094	11.615	24	0.984

**Fig 17:**
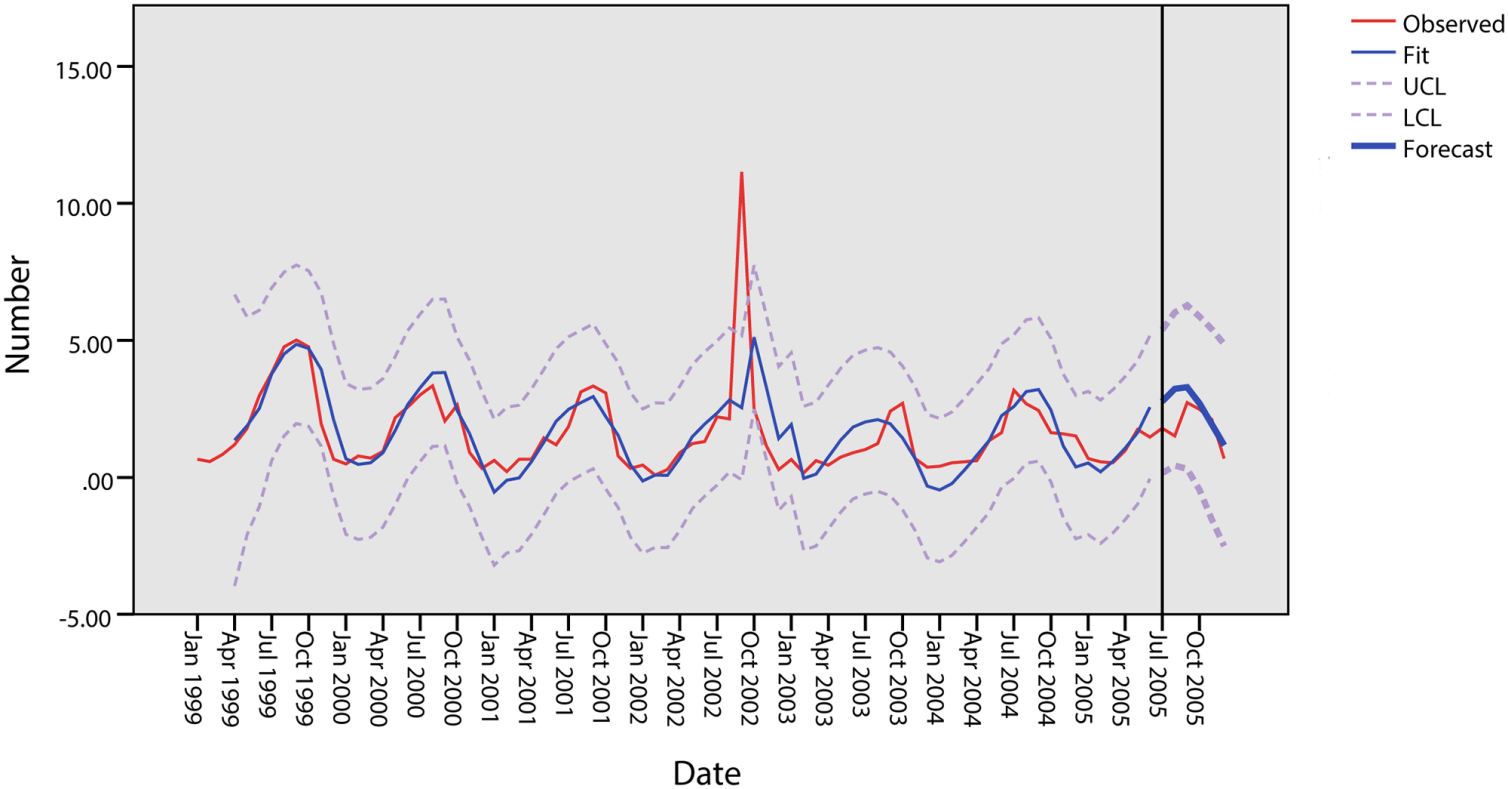
Results of the prediction model in Quzhou.

Based on the ARIMA model, this study attempts to predict cases from July to December 2005, and the result is shown in Table 8. The estimated and predicted results in Fig 17 show that the predicted data are similar to the observed data. The predicted monthly numbers of cases from July to December 2005 are 2.77, 3.23, 3.29, 2.71, 1.94 and 1.18, all of which fall within the confidence interval limits.

**Table 8:** Results of ARIMA forecasting BD incidence from July to December, 2008 in Quzhou.

Observations	Observed	Forecast	UCL	LCL
July	1.79	2.77	5.38	0.15
August	1.51	3.23	6.02	0.43
September	2.73	3.29	6.28	0.31
October	2.48	2.71	5.86	-0.45
November	2.12	1.94	5.41	-1.54
December	0.69	1.18	4.85	-2.50

Confidence Limit is 95%.

UCL: Upper Confidence Limits, LCL: Lower Confidence Limits.

## Discussion

In this study, we find that the MWVP (and particularly the MWVP^2^) has a strong relationship with BD transmission, which is a finding that has not been demonstrated in other studies. Temperature, rainfall and relative humidity have all been confirmed to be closely associated with BD transmission in previous studies. Thus, the detailed analysis of the relationship between MT, AP, RH, MWVP^2^ and BD incidence is as follows:

The correlation coefficients between MT, AP, RH, MWVP^2^ and BD incidence in Harbin and Quzhou are calculated and shown in Table 9. The *MSEs* of the prediction models using MT, AP and RH in Harbin and Quzhou are also shown in Table 9. As shown in this table, the correlation coefficients between MT, AP and BD incidence are larger than those between MWVP^2^ and BD incidence in Harbin. However, upon further analysis of the data, the *MSEs* of the prediction models using MT and AP are found to be greater than those of the model using MWVP^2^ in Harbin indicating that a high correlation coefficient does not necessarily signify a strong relationship. Though the correlation coefficients between MWVP^2^ and BD incidence are smaller, the interaction effect between MWVP and MWVP^2^ results in the highest risk of BD transmission. Moreover, the correlation coefficients between MT, AP, RH and BD incidence are all less than that between MWVP^2^ and BD incidence, and the *MSEs* of the prediction models using MT, AP and RH are all larger than those of the models in Quzhou using MWVP^2^.

**Table 9:** Correlation coefficients between MT, AP, RH, MWVP^2^ and BD incidence and *MSE* of the prediction models using MT, AP and RH respectively in Harbin and Quzhou.

	City	MT	AP	RH	MWVP^2^
Corr	Harbin	0.846**	0.781**	-0.371**	0.685**
	Quzhou	0.545**	-0.031**	-0.040**	0.694**
*MSE*	Harbin	0.296	0.416	0.977	0.251
	Quzhou	0.698	0.990	0.993	0.529

Corr means the correlation coefficients.

“**” means the significance level is 0.01.

Conversely, the correlation coefficients between MT, AP, RH and BD incidence demonstrate the differences between Harbin and Quzhou, as shown in Table 9. For example, the correlation coefficient between AP and BD incidence is 0.781** in Harbin, which indicates a strong positive correlation, whereas the coefficient is -0.031** in Quzhou, which indicates a weak negative correlation. However, MWVP^2^ demonstrates a consistent performance in both cities, including the correlation coefficients with BD incidence and the *MSEs* of the prediction models.

The t-test is applied to explore whether the *MSE* of the prediction model using MWVP^2^ is significantly different from that using MT, AP, and RH. The *MSE* of each fold is taken out as a sample in the 10-fold cross validation. Thus, we obtain 10 *MSE* values for each prediction model. The null hypothesis of the t-test is that the *MSE* of the prediction model using MWVP^2^ is equivalent to that using MT, AP and RH individually. We performed four t-tests for each city individually. The test results indicate that all of the *p* values are less than 0.1. This means that at significant level od 0.1, there is evidence to reject the null hypothesis, and the *MSE* of the prediction model using MWVP^2^ is significantly different from that using MT, AP, and RH individually.

The relationship between BD incidence and MWVP^2^ can be explained epidemiologically. Microbial metabolism and material exchange cannot happen without the inclusion of water, and microorganisms cannot live in the environment without proper humidity. Therefore, appropriately humid surroundings are necessary for biological pathogens to multiply and spread. The BD incidence has positive correlations with MAP, RH and AP. The increase in MWVP^2^ would lead to much more moisture in the air, increasing the precipitation and relative humidity. Through the analysis described in this paper, it is evident that MWVP^2^ has a strong relationship with BD incidence.

The results of previous studies are not consistent, and this is possibly due to various local climate conditions and the efficiency of the methods used. In this study, two areas with different climatic conditions are selected as the study locations, and the *MSE* of the prediction model is used as an evaluation criterion, in addition to considering the correlation between climate factors and the BD incidence. Through the analysis of the correlation coefficients between climate factors and the BD incidence described in this paper, and the *MSEs* of the prediction models, the conclusion is that the MWVP^2^ does have a stronger relationship with BD transmission than other factors, and a higher MWVP^2^ is associated with a higher incidence of BD.

## Conclusion

In this study, the effect of climate factors on BD is analysed. LASSO is used to select the relevant climate factors for the prediction of BD. Using feature selection, the MWVP^2^ is chosen as an important one. To validate the performance of the selected factors, ARIMA is applied to build the prediction model. The ARIMA model performed well, as demonstrated by the results of the experiments, and the findings indicate that the MWVP^2^ does have a significant impact on the transmission of BD. A higher MWVP^2^ is associated with a higher incidence of BD. Relevant public health strategies should be developed at an earlier stage to prevent and reduce the impact of infectious diseases that are associated with climate change.

There are two limitations in this study. Firstly, due to difficulty in determining the incidence of BD, we only obtain seven years of incidence data for each city. This paper is therefore a tentative exploration of the relationship between BD and climate factors. In subsequent studies, we will attempt to obtain more related incidence data and apply new and effective approaches to exploring the relationship between BD and climate factors. Secondly, there is only one observation point to monitor and collect weather information in each city. Harbin is located between latitudes 44°04’ and 46°40’ north and longitudes 125°42’ and 130°10’ east, and the observation point is located at latitude 45°72’ north and longitude 126°73’ east. Quzhou is located between latitudes 28°14’ and 29°30’ north and longitudes 118°01’ and 119°20’ east, and the observation point is located at latitude 28°97’ north and longitude 118°89’ east. The areas of Harbin and Quzhou span approximately 50 thousand square kilometres and 9 thousands square kilometres, respectively. Therefore, in the future works, we should evaluate if one observation point is enough to be representative of the corresponding areas in terms of climate data.
